# Survival improvement for patients with metastatic colorectal cancer over twenty years

**DOI:** 10.1038/s41698-023-00353-4

**Published:** 2023-02-13

**Authors:** Fadl A. Zeineddine, Mohammad A. Zeineddine, Abdelrahman Yousef, Yue Gu, Saikat Chowdhury, Arvind Dasari, Ryan W. Huey, Benny Johnson, Bryan Kee, Michael S. Lee, Maria Pia Morelli, Van K. Morris, Michael J. Overman, Christine Parseghian, Kanwal Raghav, Jason Willis, Robert A. Wolff, Yoshikuni Kawaguchi, Jean-Nicolas Vauthey, Ryan Sun, Scott Kopetz, John Paul Shen

**Affiliations:** 1 Department of Gastrointestinal Medical Oncology, The University of Texas MD Anderson Cancer Center, Houston, TX USA; 2 Department of Surgical Oncology, The University of Texas MD Anderson Cancer Center, Houston, TX USA; 3 Department of Biostatistics, The University of Texas MD Anderson Cancer Center, Houston, TX USA

**Keywords:** Colorectal cancer, Colorectal cancer, Prognostic markers, Chemotherapy

## Abstract

Over the past two decades of successive clinical trials in metastatic colorectal cancer (CRC), the median overall survival of both control and experimental arms has steadily improved. However, the incremental change in survival for metastatic CRC patients not treated on trial has not yet been quantified. We performed a retrospective review of 1420 patients with *de novo* metastatic CRC who received their primary treatment at the University of Texas M.D. Anderson Cancer Center (UTMDACC) from 2004 through 2019. Median OS was roughly stable for patients diagnosed between 2004 and 2012 (22.6 months) but since has steadily improved for those diagnosed in 2013 to 2015 (28.8 months), and 2016 to 2019 (32.4 months). Likewise, 5-year survival rate has increased from 15.7% for patients diagnosed from 2004 to 2006 to 26% for those diagnosed from 2013 to 2015. Notably, survival improved for patients with *BRAF*^*V600E*^ mutant as well as microsatellite unstable (MSI-H) tumors. Multivariate regression analysis identified surgical resection of liver metastasis (HR = 0.26, 95% CI, 0.19–0.37), use of immunotherapy (HR = 0.44, 95% CI, 0.29–0.67) and use of third line chemotherapy (regorafenib or trifluridine/tipiracil, HR = 0.74, 95% CI, 0.58–0.95), but not year of diagnosis (HR = 0.99, 95% CI, 0.98–1), as associated with better survival, suggesting that increased use of these therapies are the drivers of the observed improvement in survival.

## Introduction

In the past two decades remarkable progress has been made regarding the understanding of colorectal cancer (CRC) pathogenesis at a molecular level^1,2^. This molecular understanding has been translated into the first few molecularly targeted chemotherapeutic agents approved by the US Food and Drug Administration (FDA) for use in CRC, first the monoclonal antibodies cetuximab (anti-EGFR) and bevacizumab (anti-VEGF) in 2004 followed by panitumumab (anti-EGFR) in 2006^3^. This time period also saw the introduction of novel oral agents regorafenib (pan-kinase inhibitor) in 2013 and trifluridine/tipiracil (combination of cytotoxin and thymidine phosphorylase inhibitor) in 2020 for third line or greater therapy^4,5^. Oncogenic mutations in *BRAF*, a potent modulator of the MAPK pathway present in approximately 10% of CRC patients^6,7^, can now be successfully targeted with the combination of encorafenib (BRAF inhibitor) and cetuximab, which are now standard-of-care after the positive BEACON trial in 2019, and/or dabrafenib (BRAF inhibitor) and trametinib (MEK inhibitor) which were both introduced in 2014^8–11^. Microsatellite instability has also been discovered as a key biomarker predicting response to immunotherapy^12–14^, leading to the approval in 2017 of anti-PD-1 antibodies pembrolizumab and nivolumab and in 2018 combination with CTLA-4 antibody ipilimumab for treatment of MSI-H tumors^15–20^.

In addition to novel systemic therapy options, resection of liver metastases has been shown to improve long term outcomes in selected metastatic CRC populations^4,21–23^. Approximately 50% of CRC patients eventually develop liver metastases and this is often what makes CRC lethal^24,25^. Unlike many other solid tumors, resection of isolated liver metastasis can be potentially curative in CRC with 5- and 10-year survival rates of approximately 40% and 25% respectively^26–28^. For patients with favorable tumor biology (e.g., wild-types of *RAS*, *TP53*, and *SMAD4*), our group recently showed that the 5-year overall survival was approximately 70% in patients undergoing Colorectal Liver Metastases (CLM) resection^29,30^ and approximately 50% in patients undergoing simultaneous resection of CLM and extrahepatic disease^31^. However, it is estimated that only 10% to 30% of patients have limited disease that can be surgically resected with curative intent^32–36^.

In the past decade, the reported median overall survival in phase III trials in metastatic colorectal cancer has increased from approximately 16 to a 27.4 to 30 months^37,38^. This reflects an increase relative to similar trials in previously untreated metastatic CRC published from 1995 to 2008 which showed a median overall survival of 18 to 24 months^4^. Analyzing data from the Surveillance, Epidemiology, and End Results (SEER) database shows that the overall mortality rate from all stages of CRC has continually declined over the past 40 years (Supplementary Fig. 1)^39^. However, annotation from these national databases is limited and prevents more detailed analysis to identify the underlying causes of this improvement. The purpose of this study is to evaluate changes in overall survival from a large single institutional cohort and evaluate the specific clinical and/or molecular factors associated with improvement in survival.

## Results

### Patient cohort

A total of 1420 patients with *de novo* metastatic CRC treated during the 16-year period from 2004 through 2019 were identified for inclusion in the study (Table 1, Supplementary Fig. 2). There was a high degree of concordance between the institutional tumor registry database and clinical records in terms of patient demographics, date of diagnosis, history of liver resection, and vital status. The median age, location of the primary tumor, gender distribution, comorbidities and race of the population did not change significantly over the time period evaluated (Supplementary Fig. 3).

**Table 1:** Patient demographic information.

Total patients	1420 (100%)
Age in years, median [range]	55.8, [20–98]
Gender	
Male	815 (57%)
Female	605 (43%)
Race	
Black or African American	186 (13%)
Hispanic or Latino	180 (13%)
Asian	96 (7%)
White or Caucasian	902 (63%)
Comorbidities	
Cardiovascular	157 (11%)
Depression	151 (11%)
Chronic Kidney Disease	97 (7%)
Thyroid Disease	143 (10%)
Hypertension	682 (48%)
Hyperlipidemia	355 (25%)
Diabetes	279 (20%)
Therapy	
Immunotherapy	69 (5%)
Anti-EGFR	384 (27%)
Anti-BRAF	20 (1%)
Regorafenib or Tri/Ti	142 (10%)
Liver resection	
Yes	153 (11%)
No	1267 (89%)
MSI-Status	
MSS	758 (53%)
MSI-H	29 (2%)

Tri/Ti = Trifluridine/Tipiracil, MSS = Microsatellite stable, MSI-H = Microsatellite Instability High.

### Overall survival as a function of time

**Fig. 1:**
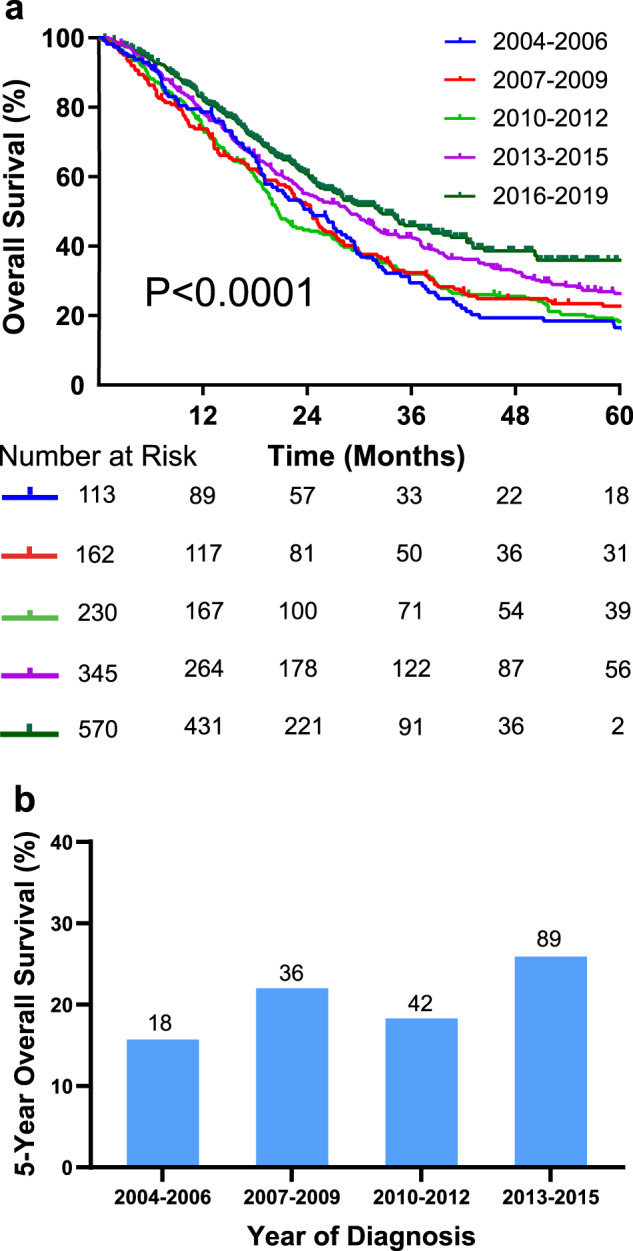
Overall survival for patients with metastatic colorectal cancer treated at M.D. Anderson Cancer Center by year of diagnosis. **a** Kaplan–Meier overall survival curves for metastatic CRC patients group by year of diagnosis, log-rank *p*-value comparing all curves <0.0001. **b** Five-year survival rate according to year of diagnosis. For 2016 to 2019, this has not yet reached.

Median overall survival remained relatively constant from 2004 through 2012 (22.6 months, 95% CI, 20.0 to 24.9 months, Fig. 1a). Following 2012, gradual improvement was observed with median overall survival of 28.8 months (95% CI, 24.5 to 33.2 months) and 32.4 months (95% CI, 27.8 to 37.0 months) for 2013 to 2015 and 2016 to 2019, respectively. Likewise, the 5-year overall survival was 19.1% for 2004 through 2012 and increased to 25.9% for 2013 to 2015 (Fig. 1b); for those diagnosed from 2016 through 2019, 5-year overall survival is not yet available.

In a univariate Cox regression analysis, year of diagnosis was associated with better overall survival with a protective effect seen for each year after 2004 (HR = 0.96, 95% CI, 0.95–0.98, 3.5 × 10^-6^, Table 2). Other factors associated with better survival included Asian race, performance of colorectal liver metastasis (CLM) resection, use of immunotherapy, and use of third line chemotherapy (regorafenib or trifluridine/tipiracil). Factors significantly associated with worse survival included chronic kidney disease (CKD), African American race, and right-sided primary tumor. Factors tested in univariate analysis but were not significantly associated with overall survival, can be seen in (Supplementary Table 1). In a multivariate analysis, CLM resection (HR = 0.26, 95% CI 0.19–0.37, *P* < 0.0001), use of immunotherapy (HR = 0.44, 95% CI 0.29–0.67, *P* = 0.0001), and use of third line chemotherapy (HR = 0.74, 95% CI, 0.58–0.95, *P* = 0.018) were associated with a better overall survival. Factors associated with worse survival in multivariate analysis included age at diagnosis (HR = 1.007, 95% CI, 1.001–1.01, *P* = 0.021), CKD (HR = 1.3, 95% CI 1.03–1.68, *P* = 0.025), African American race (HR = 1.3, 95% CI 1.04–1.54, *P* = 0.015) and right sided tumors (HR = 1.7, 95% CI 1.5–2, *P* = 4.50E–12).

**Table 2:** Significant variables from univariate and multivariate analyses.

		Univariate Analysis			Multivariate Analysis		
Variate	Reference	HR	*P*-value	95% CI	HR	*P*-value	95% CI
Liver Resection	No	0.26	2.00E-17	0.19–0.35	0.26	9.20E-16	0.19–0.37
Immunotherapy	No	0.39	5.8E-06	0.27–0.61	0.44	0.00013	0.29–0.67
Third Line Chemotherapy	No	0.69	0.0015	0.55–0.87	0.74	0.018	0.58–0.95
Race: Asian	White	0.72	0.026	0.53–0.96	0.8	0.15	0.59–1.1
Year of Diagnosis	NA	0.96	3.5E-06	0.95–0.98	0.99	0.44	0.98–1
Age at diagnosis	NA	1.012	0.000015	1.007–1.01	1.007	0.021	1.001–1.01
Chronic Kidney Disease	No	1.4	0.0029	1.1–1.8	1.3	0.025	1.03–1.68
Race: Black or African American	White	1.4	0.00047	1.2–1.7	1.3	0.015	1.04–1.54
Right sided primary tumor	Left	1.7	8.10E-12	1.4–1.9	1.7	4.50E-12	1.5–2

Only variables significant in univariable analysis were included in the multivariable model. Regression Formula: OS ~ Age at Diagnosis + Primary Tumor Side + Immunotherapy + Third Line Treatment + Race + Liver Resection + Chronic Kidney Disease + Year of diagnosis.

### Hepatic colorectal liver metastases resection

**Fig. 2:**
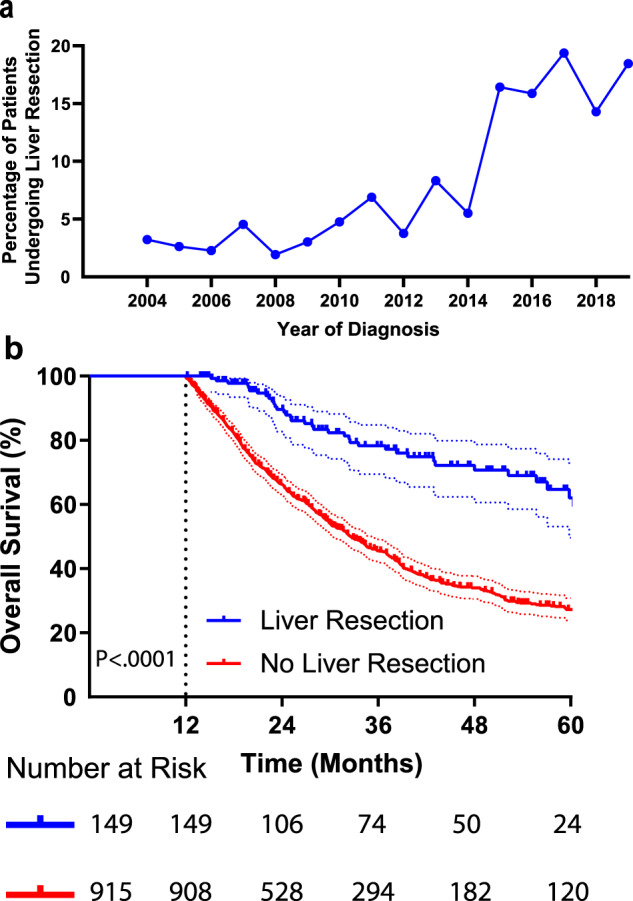
Hepatic Resection of Liver Metastasis. **a** Percentage of patients undergoing liver resection by date of diagnosis, note increase after 2014. **b** Overall survival by landmark analysis of patients with metastatic colorectal cancer diagnosed between 2004 and 2019, error bars represent 95% CI, Log-rank *p* < 0.0001.

One hundred fifty-three patients (10.8%) in the cohort underwent hepatic Colorectal Liver Metastases (CLM) resection; the fraction of patients undergoing CLM resection increased from 2004 to 2019 (Fig. 2a). Before 2014, only 5% of patients had CLM resection, however after that the frequency increased sharply in 2015, peaking at 19.4% in 2017. To account for immortal time bias, landmark analysis was used to evaluate the impact of hepatic resection on survival^40^. Using a 12-month landmark, the 5-year survival rate for patients who had undergone hepatic metastasis resection was 58.3%, compared with 27.0% for patients without resection; median overall survival for these two groups was 74.3 months (95% CI, 58.5 to 90.0 months) and 32.6 months (95% CI, 30.1 to 35.2 months), respectively with HR of 0.33 (95% CI, 0.22 to 0.41, *P* < 0.0001, Fig. 2b). Since a landmark time was not pre-specified, landmark analysis was also performed with intervals of 6, 18, and 24 months all of which showed that patients who underwent CLM resection had superior overall survival (Supplementary Fig. 4).

### Chemotherapy utilization

**Fig. 3:**
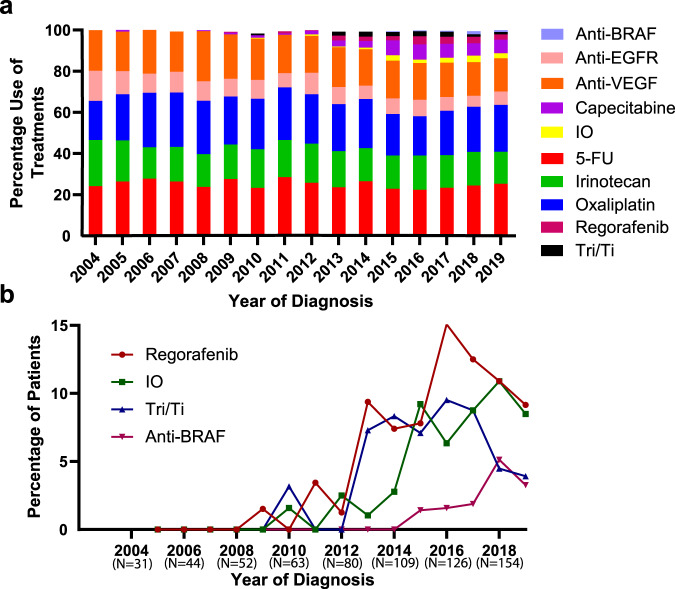
Chemotherapy utilization changes over time. **a** The percentage of each chemotherapy administered to patients between 2004 and 2019, binned by year, note use of novel chemotherapeutics increased after 2012. **b** Percentage of patients treated with novel drugs, IO Immunotherapy, Tri/TI Trifluridine/Tipiracil.

To estimate the impact of changes in chemotherapy and to exclude the effects of hepatic CLM resection, overall survival analyses were repeated after removing patients who underwent hepatic resection. Median overall survival remained relatively constant from 2004 through 2012 (22 months, 95% CI, 19.8 to 24.175 months, Supplementary Fig. 5). Following 2012, gradual improvement was observed with median overall survival of 28 months (95% CI, 23.8 to 32 months) and 28.3 months (95% CI, 24.1 to 32.3 months) for 2013 to 2015 and 2016 to 2019 respectively (Supplementary Fig. 5). Review of institutional pharmacy records shows a temporal association of these improvements with the adoption of additional medical treatment options beyond fluorouracil, irinotecan, oxaliplatin, bevacizumab, and cetuximab (Fig. 3a). Prior to 2012, therapies such as regorafenib, immunotherapy (IO), trifluridine/tipiracil (Tri/Ti), and BRAF inhibitors were not available. However, after 2012, the percentage of patients receiving these therapies started to increase with use of regorafenib, immune therapy, Tri/Ti, and BRAF inhibitors reaching to 15%, 10.9%, 9.5%, and 5.1% of patients, respectively (Fig. 3b).

### Molecular biomarkers

**Fig. 4:**
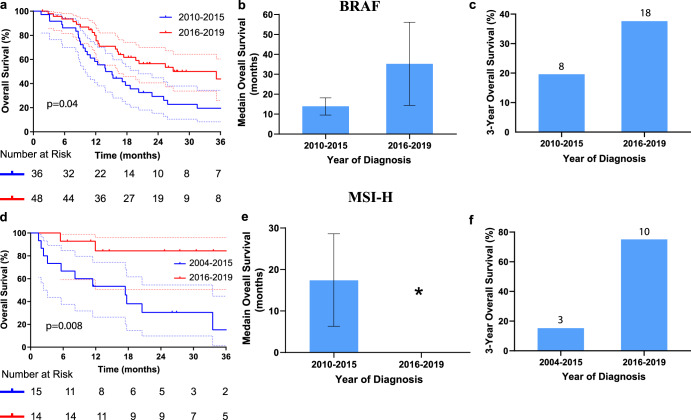
Overall survival for patients with metastatic colorectal cancer with either *BRAF* mutation or microsatellite instability. **a** Kaplan–Meier overall survival curves for patients with *BRAF* mutation before and after 2014, error bars represent 95% CI, log-rank *p* = 0.04. Median overall survival (**b**) and three-year survival rate (**c**) for patients with *BRAF* mutation, note improvement after 2015. Kaplan–Meier overall survival curve (**d**), median overall survival, error bars represent 95% CI, log-rank *p* = 0.008 (**e**) and three-year survival rate (**f**) for MSI-H patients, note improvement after 2015 where median overall survival is not-yet-reached. Number of patients indicated above bar plot.

The utilization of biomarker testing changed over time. To overcome this limitation, survival analysis in molecularly defined subgroups was limited to time periods where majority of patients were tested. Additionally, 3-year overall survival was taken for comparison instead of 5-year due to limited 5-year follow-up for these patients. Concerning patients with *BRAF* mutation, there was significant improvement in median overall survival when comparing 2010 through 2015 (13.9 months, 95% CI, 9.5 to 18.2 months) to 2016 to 2019 (35.2 months, 95% CI, 14.4 to 56.1 months) (HR = 0.54, 95% CI 0.3 to 1, *P* = 0.04, Fig. 4a, b). Likewise, the 3-year overall survival rate was 19.6% for 2010 through 2015 and increased to 37.6% for 2016 through 2019 (Fig. 4c). As for MSI-H, the difference in median overall survival time was also significant (HR = 0.14, 95% CI 0.04 to 0.42, *P* = 0.003, Fig. 4d). For patients diagnosed from 2004 through 2015, median overall survival time was 17.4 months (95% CI, 6.3 to 28.6 months) vs. not-yet-reached for those diagnosed from 2016 through 2019 (Fig. 4e). 3-year overall survival increased from 15.2% for patients diagnosed from 2004 through 2015 to 75% for patients diagnosed from 2016 through 2019 (Fig. 4f). Patients with *KRAS* mutant tumors had worse survival relative to *KRAS* wildtype (median OS 26.8 vs 37.1 months, HR = 1.3, *p*-value = 0.0007, Supplementary Fig. 6).

### Primary tumor sidedness

**Fig. 5:**
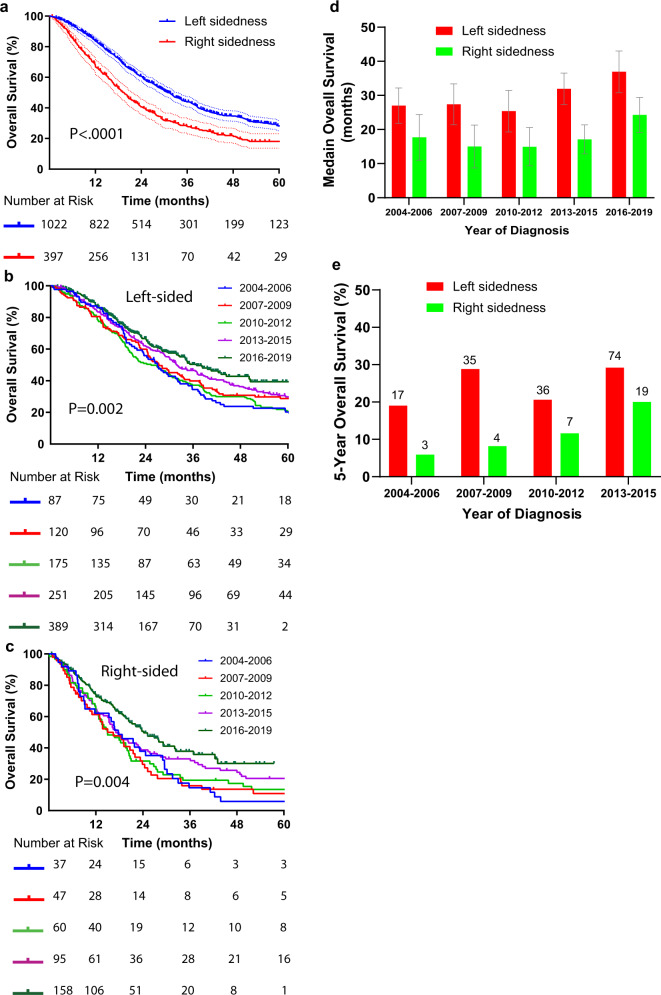
Overall survival for patients with metastatic colorectal according to primary tumor location. **a** Kaplan–Meier overall survival curves comparing patients based on primary site location, log-rank *p* < 0.0001. Kaplan–Meier overall survival curves showing the change in overall survival of patients with left (**b**) and right (**c**) sided tumors. **d** Median overall survival of patients according to tumor location binned by time of diagnosis. **e** Five-year survival rate according to tumor location binned by time of diagnosis. For 2016 to 2019, this has not yet reached.

For patients diagnosed from 2004 through 2019, the overall survival rate for patients with primary tumors in the left colon was 28% compared to 17.5% for those with right sided tumors (HR: 0.63, 95% CI, 0.54 to 0.71, *P* < 0.0001, Fig. 5a). The change in survival over time was similar for patients with either left or right sided tumors, with left sided tumors consistently showing better survival for each time interval evaluated (Fig. 5b–d). Likewise, the 5-year overall survival rate was also consistently better for patients with left-sided tumors (Fig. 5e).

## Discussion

This retrospective study highlights the gradual improvement in overall survival for patients with metastatic CRC over the last twenty years. This finding is consistent with the trend seen in the national SEER database^39^, but these single institution data offer greater granularity by including important data regarding treatment history as well as pathologic and molecular biomarkers. The results of univariate and multivariate regression analyses suggest that the primary drivers of this survival improvement are the increased utilization of CLM resection, the use of immunotherapy for MSI-H tumors, and the use of third line chemotherapy. Resection of hepatic metastases increased significantly after 2014, and in the last few years has stabilized at approximately 20% of metastatic CRC patients. This percentage is similar to other recent retrospective surgical case series, as is the median overall survival of over six years in resected patients according to reports in early 2000s^4,32,41–45^ and reports after 2010^23,46,47^. The possibility of long term disease free survival underscores the importance of considering hepatic resection as a treatment option for patients with metastatic CRC^23,48,49^. However, proper selection of patients most likely to benefit from CLM resection is important and non-trivial^50–52^; at UTMDACC, it is standard practice to present all potential CLM resection candidates at a multi-disciplinary tumor board to reach consensus regarding resectability, potential neo-adjuvant chemotherapy, and potential non-surgical local therapy^53^.

Although excellent outcomes were observed in patients undergoing CLM resection, the fact that improvement in survival was seen when CLM patients were removed from analysis (Supplementary Fig. 5) indicates that other factors are contributing to survival improvement. Multivariate regression analysis indicates that use of immunotherapy, which was rare prior to 2015, was also a key driver of the observed improvement in survival. In the era of immunotherapy, the median OS for patients with MSI-H tumors was not-yet-reached (Fig. 4d), suggesting that similar to CLM, many of these patients will experience long-term disease-free survival. Although only CLM resection, use of immune therapy, and use of third line chemotherapy remained significant in multivariate analysis, there are likely multiple other factors contributing to the survival improvement, some of which may not have been well captured by our study. Improvements in the radiographic staging of CRC including higher resolution CT scanning, as well as use of magnetic resonance imaging (MRI) with diffusion weighted imaging (DWI), and integrated positron-emission tomography (PET)/CT allow for better risk stratification^54^ and ultimately aid in the selection of the optimal therapy for each patient. Advances in molecular biomarkers, including the discovery that Ras mutant tumors do not respond to anti-EGFR antibodies^55^ and are more likely to recur after CLM^47^, have similarly aided optimizing the therapy for each patient. Improvements in supportive care, including the management of comorbid diseases, also likely contributed to survival improvements but were not well captured in our study. Similarly, data on socioeconomic status, which has been associated with survival in cancer patients^56^, was not available. It is also possible that socioeconomic status contributes to the worse outcomes seen in African American patients and better outcomes seen in Asian patients (Table 2), although differences intrinsic differences in tumor genetics may also contribute^57^.

As a retrospective, single institutional study there are several inherent limitations to study design. With regards to the retrospective collection of data, the completeness of documentation was a factor outside of our control, for this reason only patients who received their chemotherapy at UTMDACC were included in the study as documentation for many patients seen only as consults or second opinions was incomplete. Incomplete documentation of chemotherapy treatments or stage in the earliest years of this study is the mostly likely reason less patients were included from those years, however there was no indication that this restriction biased the early part of the cohort in terms of performance status or comorbidity, race, or other demographic features. Finally, we did not capture if patients underwent tumor resection in extrahepatic sites, nor interventional radiology ablative procedures or Selective Internal Radiation Therapy (SIRT)^58,59^. UTMDACC is a tertiary referral center, with a significant fraction of patients traveling from out of state for treatment (Supplementary Fig. 7), which has a tendency to skew the patient population to higher socioeconomic status, better performance status, and younger age relative to the broader metastatic CRC population in the United States. However, patients in community cancer centers have access to the same FDA approved drugs as those at an academic center, and it is increasingly being recognized that it is critical to include liver surgeons in community oncology tumor boards to identify potentially resectable candidates^60,61^. Therefore, we feel that these data are relevant to so called “real world” metastatic CRC patients in both community and academic settings.

In summary, the prognosis of patients diagnosed with metastatic CRC has improved significantly over the last twenty years. However, even with these improvements only a minority of patients will survive five years from their time of diagnosis, highlighting the critical need for continued research to develop better treatments for what remains a lethal disease. Clinically, these data underscore the importance of identifying potential candidates for immune therapy and CLM resection.

## Methods

### Institutional patient identification

This study was approved by the UTMDACC Institutional Review Board, protocol 09-0373; a waiver of informed consent was granted per USA federal regulation 45 CFR 46.116(f) (Common Rule) given minimal risk to patients. Adult patients diagnosed with metastatic CRC were identified from the electronic health record (EHR) at the University of Texas M.D. Anderson Cancer Center (UTMDACC) using the Foundry software system (Palantir Technologies, Denver, CO). Patients diagnosed between January 1, 2004, and December 31, 2019, were selected for study, with follow-up until April 15, 2021. Only patients with confirmed adenocarcinoma of colon and/or rectum and *de novo* metastatic disease were included in the study. Patients who underwent resection of hepatic metastases were identified using a prospectively maintained surgical database. Patients who visited just for consultation and did not receive their treatment at UTMDACC were excluded (Supplementary Fig. 2). Patient characteristics can be seen in Table 1.

Vital status was determined through clinical follow-up, search of administrative death indices, and follow-up correspondence to patients. Pharmacy databases were used to extract chemotherapy administration details in an automated fashion using the Foundry system. The percentage of each chemotherapy administered in a given year was calculated with respect to the total chemotherapy treatments given to all metastatic CRC patients in the study in the same year. To verify information extracted via the Foundry system and tumor registry, 5% of the available patients were randomly selected and charts reviewed manually to confirm the presence of metastatic disease, tumor histology, diagnosis date, vital status, chemotherapy administered, and history of liver resection.

### Statistical analysis

Cox Proportional hazard model was first fit by univariates analysis; a *p*-value < 0.05 was considered significant in the model. Significant factors were included for further multivariate analysis. Variables included were: Age at diagnosis, Anti-BRAF, Anti-EGFR, Cardiovascular, Chronic Kidney Disease, Depression, Diabetes, Gender, Hyperlipidemia, Hypertension, Immunotherapy, Liver Resection, Third Line Treatment, Race, Primary Tumor Sidedness, Thyroid Disease, Year of Diagnosis. A *P* < 0.05 was considered significant. Overall survival analysis was fit to 1420 patients, and Kaplan–Meier survival curves were generated. Comparison of different groups was performed using the log-rank test; *p*-value < 0.05 was considered significant. For analysis of the impact of hepatic resection on survival, the length of survival is known to impact the possibility that patients will undergo hepatic resection, thereby inducing a bias in favor of resection using traditional survival methods^40^. Hence, a landmark analysis was used to decrease bias induced by including various events that happens after the baseline hazard models^4,62,63^. The landmark time used was 6, 12, 18, and 24 months and the analysis included only patients alive after any of those points, and compared the survival outcome between patients who had and had not undergone resection in said months. A landmark analysis was done to evaluate the impact of resection after controlling for 6, 12, 18, and 24 months of diagnosis^40^. In addition, Kaplan–Meier curves were plotted after removing patients who underwent partial resection to assess outcomes other than surgery on survival. All analyses were performed using SPSS version 26.0 (SPSS, Chicago, IL), GraphPad Prism version 8.0 (GraphPad; La Jolla, CA), and R version 4.0.1.

### Molecular data

Molecular testing was performed at MD Anderson’s College of American Pathologists (CAP) accredited and Clinical Laboratory Improvement Amendments (CLIA) certified molecular diagnostics laboratory. PCR-based next generation sequencing (NGS) was used to test for mutations in the coding sequence of 134 genes and copy number variations (CNV) in 47 genes as previously described^64^ using GRCh37/hg19 as reference sequence. Microsatellite status was determined by immunohistochemistry evaluation for mismatch repair proteins MLH1, MSH2, MSH6, and PMS2 per standard criteria^65^.

### Reporting summary

Further information on research design is available in the Nature Research Reporting Summary linked to this article.

## Supplementary information

Supplemental Material

REPORTING SUMMARY

## Data Availability

The datasets generated during and/or analyzed during the current study are not publicly available to maintain compliance with IRB protocol. Anonymized data are available for non-commercial use from corresponding author upon request pending data usage agreement and/or IRB-approved collaboration.

## References

[1] Singh MP, Rai S, Pandey A, Singh NK, Srivastava S (2021). Molecular subtypes of colorectal cancer: an emerging therapeutic opportunity for personalized medicine. Genes Dis..

[2] Sjöblom T (2006). The consensus coding sequences of human breast and colorectal cancers. Science.

[3] U. S. Food and Drug Administration. https://www.accessdata.fda.gov/scripts/cder/daf/index.cfm. Accessed November 17, 2021.

[4] Kopetz S (2009). Improved survival in metastatic colorectal cancer is associated with adoption of hepatic resection and improved chemotherapy. J. Clin. Oncol..

[5] Van Cutsem E (2016). ESMO consensus guidelines for the management of patients with metastatic colorectal cancer. Ann. Oncol. Off. J. Eur. Soc. Med. Oncol. / ESMO.

[6] Davies H (2002). Mutations of the BRAF gene in human cancer. Nature.

[7] Clarke CN, Kopetz ES (2015). BRAF mutant colorectal cancer as a distinct subset of colorectal cancer: clinical characteristics, clinical behavior, and response to targeted therapies. J. Gastrointest. Oncol..

[8] Molina-Cerrillo J (2020). BRAF mutated colorectal cancer: new treatment approaches. Cancers.

[9] Corcoran RB (2015). Combined BRAF and MEK inhibition with dabrafenib and trametinib in BRAF V600-mutant colorectal cancer. J. Clin. Oncol..

[10] Bendell JC (2014). Efficacy and tolerability in an open-label phase I/II study of MEK inhibitor trametinib (T), BRAF inhibitor dabrafenib (D), and anti-EGFR antibody panitumumab (P) in combination in patients (pts) with BRAF V600E mutated colorectal cancer (CRC). J. Clin. Oncol..

[11] Kopetz S (2019). Encorafenib, binimetinib, and cetuximab in BRAF V600E–mutated colorectal cancer. N. Engl. J. Med..

[12] Loeb LA, Springgate CF, Battula N (1974). Errors in DNA replication as a basis of malignant changes. Cancer. Res.

[13] Jiricny J (2006). The multifaceted mismatch-repair system. Nat. Rev. Mol. Cell Biol..

[14] Battaglin F, Naseem M, Lenz HJ, Salem ME (2018). Microsatellite instability in colorectal cancer: overview of its clinical significance and novel perspectives. Clin. Adv. Hematol. Oncol..

[15] André T (2020). Pembrolizumab in microsatellite-instability–high advanced colorectal cancer. N. Engl. J. Med..

[16] Le DT (2015). PD-1 blockade in tumors with mismatch-repair deficiency. N. Engl. J. Med.

[17] Le DT (2017). Mismatch repair deficiency predicts response of solid tumors to PD-1 blockade. Science.

[18] Overman MJ (2017). Nivolumab in patients with metastatic DNA mismatch repair-deficient or microsatellite instability-high colorectal cancer (CheckMate 142): an open-label, multicentre, phase 2 study. Lancet Oncol..

[19] Le DT (2020). Phase II open-label study of pembrolizumab in treatment-refractory, microsatellite instability-high/mismatch repair-deficient metastatic colorectal cancer: KEYNOTE-164. J. Clin. Oncol..

[20] Overman MJ (2018). Durable Clinical Benefit With Nivolumab Plus Ipilimumab in DNA Mismatch Repair-Deficient/Microsatellite Instability-High Metastatic Colorectal Cancer. J. Clin. Oncol..

[21] Tomlinson JS (2007). Actual 10-year survival after resection of colorectal liver metastases defines cure. J. Clin. Oncol. Off. J. Am. Soc. Clin. Oncol..

[22] Abdalla EK (2004). Recurrence and outcomes following hepatic resection, radiofrequency ablation, and combined resection/ablation for colorectal liver metastases. Ann. Surg..

[23] Kawaguchi Y (2022). Improved survival over time after resection of colorectal liver metastases and clinical impact of multigene alteration testing in patients with metastatic colorectal cancer. J. Gastrointest. Surg..

[24] van der Pool AE (2012). Trends in incidence, treatment and survival of patients with stage IV colorectal cancer: a population-based series. Colorectal Dis..

[25] Wilkes GM (2011). Metastatic colorectal cancer: management challenges and opportunities. Oncol. (Williston Park).

[26] Kanas GP (2012). Survival after liver resection in metastatic colorectal cancer: review and meta-analysis of prognostic factors. Clin. Epidemiol..

[27] Adams RB (2013). Selection for hepatic resection of colorectal liver metastases: expert consensus statement. HPB.

[28] Kawaguchi Y (2020). A new surveillance algorithm after resection of colorectal liver metastases based on changes in recurrence risk and ras mutation status. J. Natl Compr. Cancer Netw. Jnccn..

[29] Kawaguchi Y (2021). Genomic sequencing and insight into clinical heterogeneity and prognostic pathway genes in patients with metastatic colorectal cancer. J. Am. Coll. Surg..

[30] Kawaguchi Y (2019). Mutation status of RAS, TP53, and SMAD4 is superior to mutation status of RAS alone for predicting prognosis after resection of colorectal liver metastases. Clin. Cancer Res. Off. J. Am. Assoc. Cancer Res..

[31] Lillemoe HA (2022). RAS/TP53 co-Mutation is Associated with Worse Survival after Concurrent Resection of Colorectal Liver Metastases and Extrahepatic Disease. Ann. Surg..

[32] Folprecht G, Grothey A, Alberts S, Raab HR, Kohne CH (2005). Neoadjuvant treatment of unresectable colorectal liver metastases: correlation between tumour response and resection rates. Ann. Oncol..

[33] Bismuth H (1996). Resection of nonresectable liver metastases from colorectal cancer after neoadjuvant chemotherapy. Ann. Surg..

[34] Garden OJ (2006). Guidelines for resection of colorectal cancer liver metastases. Gut.

[35] Stangl R, Altendorf-Hofmann A, Charnley RM, Scheele J (1994). Factors influencing the natural history of colorectal liver metastases. Lancet.

[36] Nordlinger B (2009). Combination of surgery and chemotherapy and the role of targeted agents in the treatment of patients with colorectal liver metastases: recommendations from an expert panel. Ann. Oncol..

[37] Cremolini C (2020). Upfront FOLFOXIRI plus bevacizumab and reintroduction after progression versus mFOLFOX6 plus bevacizumab followed by FOLFIRI plus bevacizumab in the treatment of patients with metastatic colorectal cancer (TRIBE2): a multicentre, open-label, phase 3, randomised, controlled trial. Lancet Oncol..

[38] Venook AP (2017). Effect of first-line chemotherapy combined with cetuximab or bevacizumab on overall survival in patients With KRAS wild-type advanced or metastatic colorectal cancer: a randomized clinical trial. JAMA.

[39] *Surveillance E, and End Results (SEER) Program* (www.seer.cancer.gov) SEER*Stat Database. Incidence - SEER 18 Regs Research Data + Hurricane Katrina Impacted Louisiana Cases, Nov 2017 Sub (1973-2015 varying) - Linked To County Attributes - Total U.S., 1969-2016 Counties, National Cancer Institute, DCCPS, Surveillance Research Program, released April 2018, based on the November 2017 submission.

[40] Farr AM, Foley K (2013). Landmark analysis to adjust for immortal time bias in oncology studies using claims data linked to death data. Value Health.

[41] Choti MA (2002). Trends in long-term survival following liver resection for hepatic colorectal metastases. Ann. Surg..

[42] Figueras J (2001). Resection rate and effect of postoperative chemotherapy on survival after surgery for colorectal liver metastases. Br. J. Surg..

[43] Abdalla EK (2004). Recurrence and outcomes following hepatic resection, radiofrequency ablation, and combined resection/ablation for colorectal liver metastases. Ann. Surg..

[44] Pawlik TM (2005). Effect of surgical margin status on survival and site of recurrence after hepatic resection for colorectal metastases. Ann. Surg..

[45] Adam R (2007). Developing strategies for liver metastases from colorectal cancer. Semin Oncol..

[46] Kawaguchi Y (2019). Conditional recurrence-free survival after resection of colorectal liver metastases: persistent deleterious association with RAS and TP53 co-mutation. J. Am. Coll. Surg..

[47] Chun YS (2019). Deleterious effect of RAS and evolutionary high-risk TP53 double mutation in colorectal liver metastases. Ann. Surg..

[48] Nishioka Y (2022). Neither surgical margin status nor somatic mutation predicts local recurrence after R0-intent resection for colorectal liver metastases. J. Gastrointest. Surg..

[49] Kawaguchi Y (2021). Contour prognostic model for predicting survival after resection of colorectal liver metastases: development and multicentre validation study using largest diameter and number of metastases with RAS mutation status. Br. J. Surg..

[50] Ignatavicius P (2020). Choices of therapeutic strategies for colorectal liver metastases among expert liver surgeons: a throw of the dice?. Ann. Surg..

[51] Hackl C (2014). Treatment of colorectal liver metastases in Germany: a ten-year population-based analysis of 5772 cases of primary colorectal adenocarcinoma. BMC Cancer.

[52] Bowles BJ (2001). Safety and efficacy of radiofrequency thermal ablation in advanced liver tumors. Arch. Surg..

[53] Adam R (2015). Managing synchronous liver metastases from colorectal cancer: a multidisciplinary international consensus. Cancer Treat. Rev..

[54] Goiffon RJ, O’Shea A, Harisinghani MG (2021). Advances in radiological staging of colorectal cancer. Clin. Radiol..

[55] Misale S, Di Nicolantonio F, Sartore-Bianchi A, Siena S, Bardelli A (2014). Resistance to Anti-EGFR therapy in colorectal cancer: from heterogeneity to convergent evolution. Cancer Discov..

[56] Woods LM, Rachet B, Coleman MP (2006). Origins of socio-economic inequalities in cancer survival: a review. Ann. Oncol..

[57] Augustus GJ, Ellis NA (2018). Colorectal cancer disparity in african americans: risk factors and carcinogenic mechanisms. Am. J. Pathol..

[58] Glehen O (2004). Cytoreductive surgery combined with perioperative intraperitoneal chemotherapy for the management of peritoneal carcinomatosis from colorectal cancer: a multi-institutional study. J. Clin. Oncol..

[59] McAfee MK (1992). Colorectal lung metastases: results of surgical excision. Ann. Thorac. Surg..

[60] Vega EA (2021). Failure to cure patients with colorectal liver metastases: the impact of the liver surgeon. Ann. Surg. Oncol..

[61] Raoof M (2020). Systematic failure to operate on colorectal cancer liver metastases in California. Cancer. Med..

[62] Anderson JR, Cain KC, Gelber RD (1983). Analysis of survival by tumor response. J. Clin. Oncol..

[63] Burzykowski TMG, Buyse M (2004). The validation of surrogate end points by using data from randomized clinical trials: a case-study in advanced colorectal cancer. J. R. Stat. Soc. A..

[64] Luthra R (2017). A targeted high-throughput next-generation sequencing panel for clinical screening of mutations, gene amplifications, and fusions in solid tumors. J. Mol. Diagn..

[65] Umar A (2004). Revised bethesda guidelines for hereditary nonpolyposis colorectal cancer (lynch syndrome) and microsatellite instability. JNCI: J. Natl. Cancer Inst..

