# Supplementary material for: Survival improvement for patients with metastatic colorectal cancer over twenty years
Source: NPJ Precis Oncol. 2023 Feb 13;7:16. doi: 10.1038/s41698-023-00353-4 (PMC9925745; doi:10.1038/s41698-023-00353-4)
Supplement: Supplementary file 1 — Supplemental Material [file 41698_2023_353_MOESM1_ESM.pdf]

Supplementary Figure 1. Mortality rate.

Supplementary Figure 2. CONSORT diagram.

Supplementary Figure 3. Patient characteristics over time.

Supplementary Figure 4. Landmark analysis of effect of liver metastasis resection.

Supplementary Figure 5. Overall survival for patients with metastatic colorectal cancer who did not undergo liver resection.

Supplementary Figure 6. Overall survival stratified by *KRAS* mutation status.

Supplementary Figure 7. Distribution of patient's Zip code over time.

Supplementary Table 1. Factors tested, but not significantly associated with Overall Survival (OS) in univariate regression analysis.

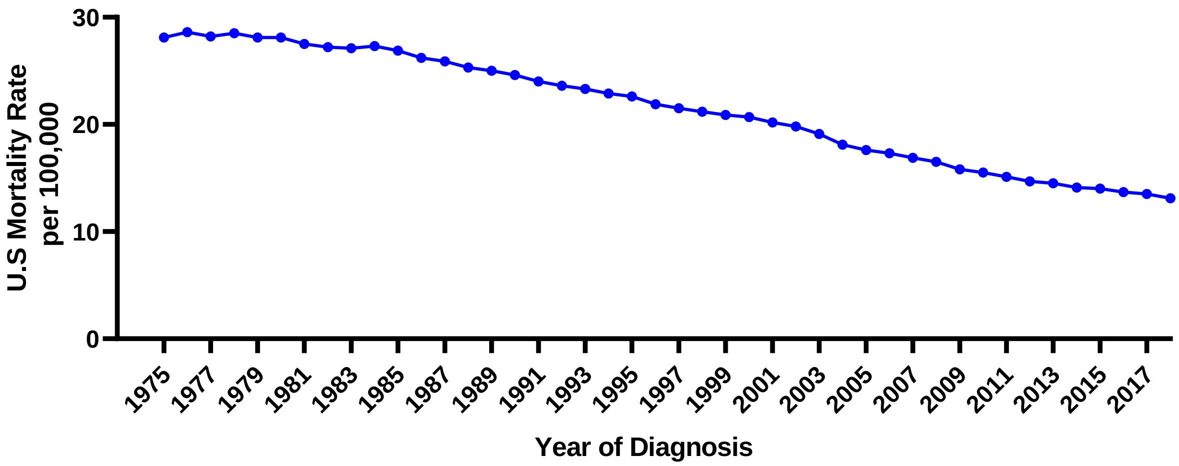

Supplementary Figure 1. Mortality Rate. Mortality Rate per 100,000 for all CRC stages in the United State by year, data from SEER. Note a persistent decreasing trend throughout the time period.

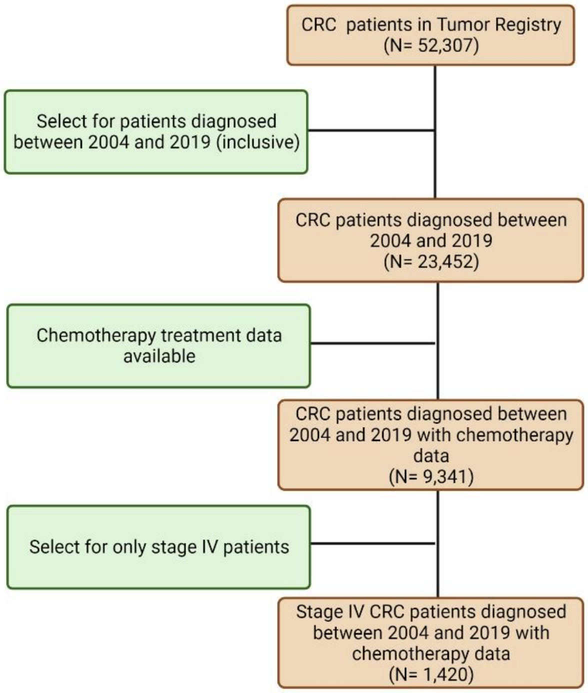

Supplementary Figure 2. CONSORT diagram showing identification of metastatic CRC cohort.

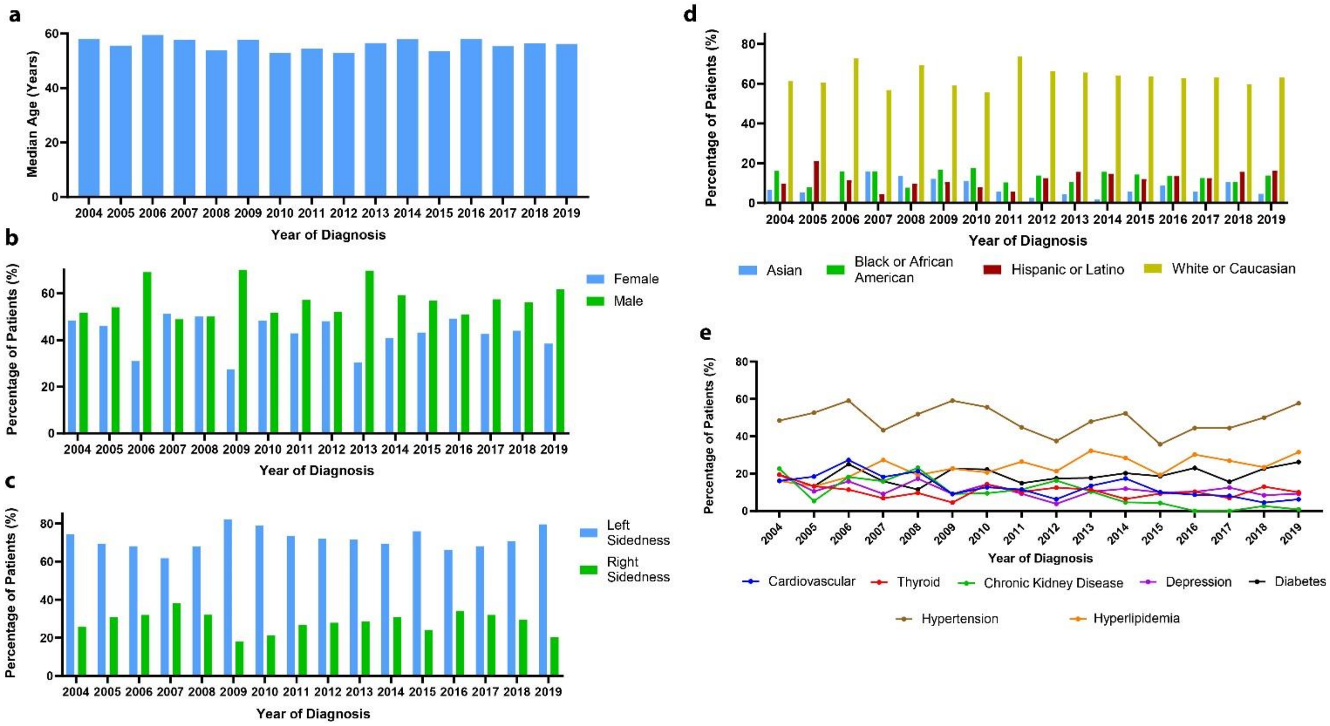

Supplementary Figure 3. Patient characteristics over time. (a) Median age at time of diagnosis binned by year of diagnosis. **(b)** Gender distribution binned by year of diagnosis. **(c)** Tumor location binned by year of diagnosis. **(d)** Race distribution binned by year of diagnosis. **(e)** Comorbidities distribution over the years.

a

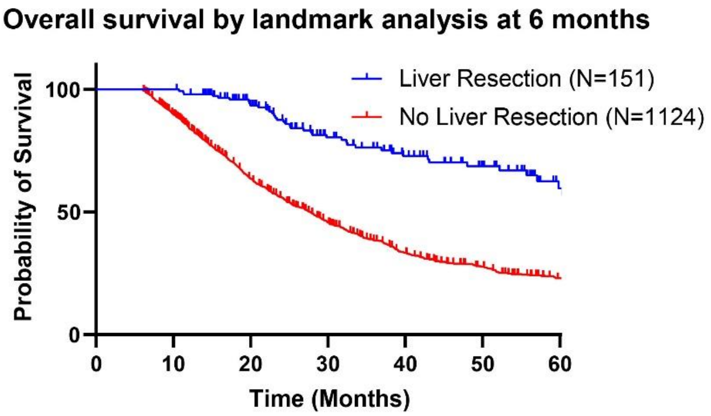

b

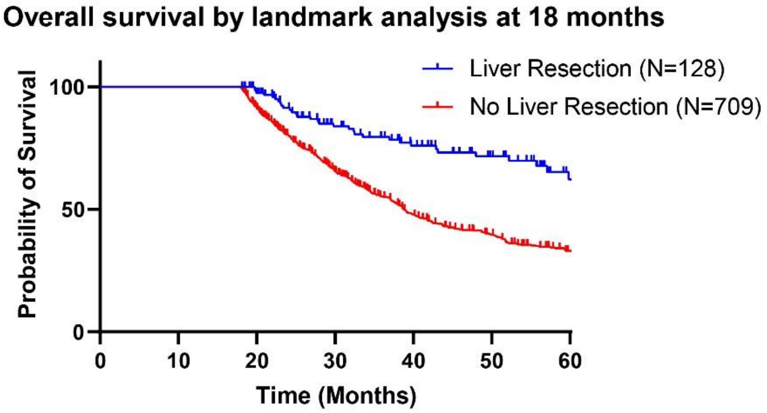

c

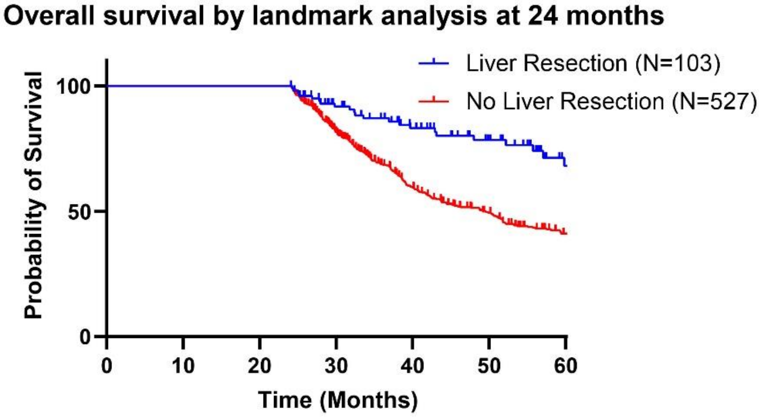

Supplementary Figure 4. Landmark Analysis. Overall survival by landmark analysis at 6 (**a**), 18 (**b**) and 24 months (**c)**. Median overall survival of patients who had liver resection is consistently greater than that of those who did not undergo liver resection. At 6 months, the median overall survival was 74.7 months for liver resection group vs 27.7 months for unresected group (HR = 0.29, 95% CI, 0.24 to 0.35; P <0.0001). At 18 months, the median overall survival was not-yet-reached for liver resection group vs. 38.8 months for unresected group (HR = 0.40 95% CI, 0.31 to 0.51; P <0.0001). At 24 months, the median overall survival was not-yet-reached for liver resection group vs. 49.2 months for unresected group (HR = 0.40 95% CI, 0.3 to 0.54; P <0.0001).

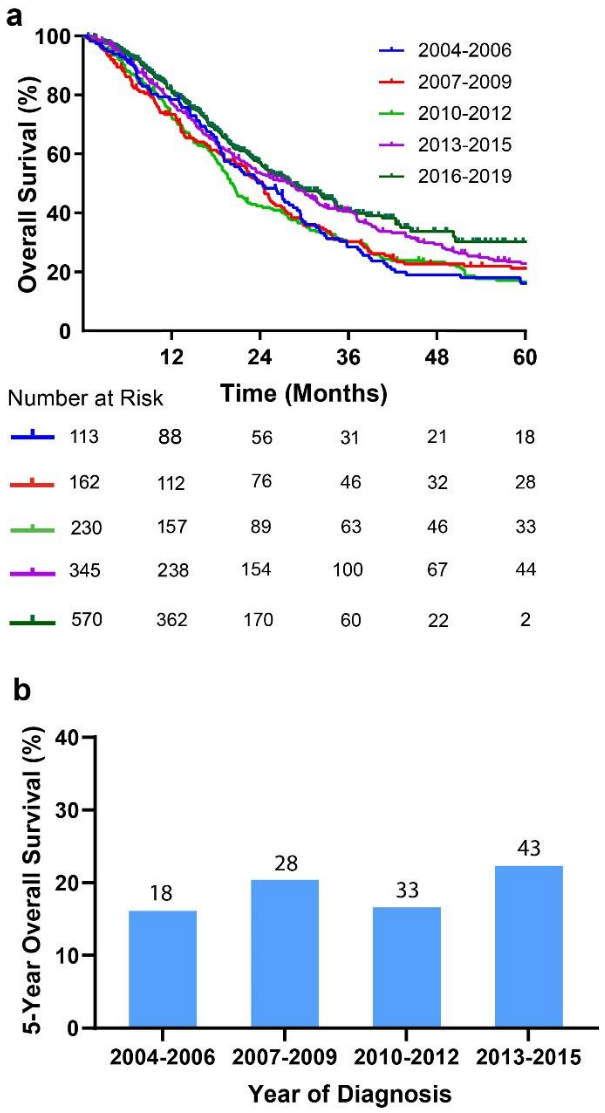

Supplementary Figure 5. Overall survival for patients with metastatic colorectal cancer who did not undergo liver resection. (a) Kaplan-Meier overall survival curves for metastatic CRC patients grouped by year of diagnosis, log-rank p-value comparing all curves = 0.003. **(b)** Five-year survival rate grouped by year of diagnosis. For 2016 to 2019, this has not reached yet.

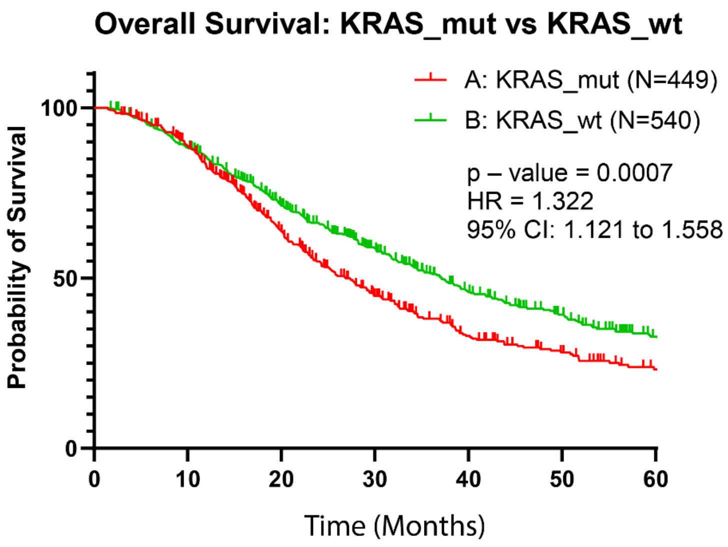

Supplementary Figure 6. Overall survival stratified by *KRAS* mutation status

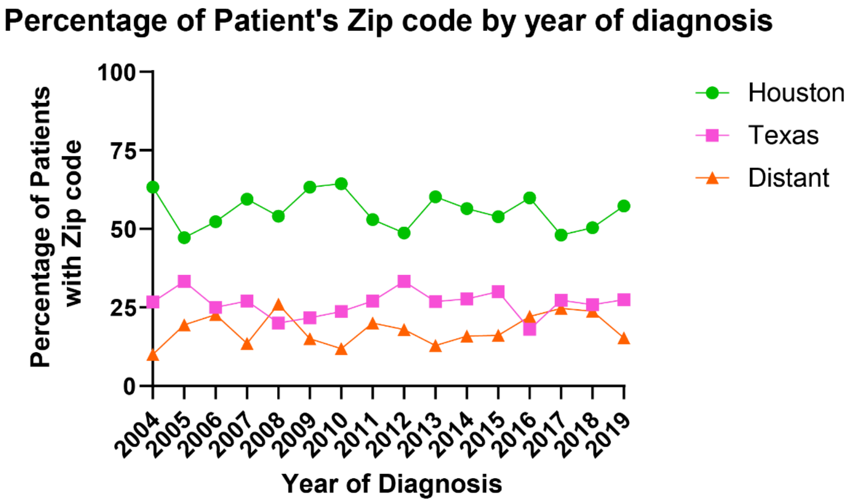

Supplementary Figure 7. Distribution of patient’s zip codes over time.

		Univariate Analysis		
**Variate**	**Reference**	**HR**	**P-value**	**95 % CI**
Depression	No	0.85	0.14	0.69-1.1
Race: Hispanic or Latino	White	0.9	0.3	0.73-1.1
Anti-EGFR Therapy	No	0.93	0.35	0.81-1.1
Diabetes	No	0.95	0.51	0.8-1.1
Hyperlipidemia	No	0.97	0.65	0.83-1.1
Cardiovascular Disease	No	0.98	0.83	0.78-1.2
Hypertension	No	0.98	0.73	0.86-1.1
Male Gender	Female	0.99	0.92	0.87-1.1
Thyroid Disease	No	0.99	0.92	0.8-1.2
Anti-BRAF Therapy	No	1	0.94	0.55-1.9

Supplementary Table 1. Factors tested, but not significantly associated with Overall Survival (OS) in univariate regression analysis. Cardiovascular Disease = Atherosclerosis, Cardiomyopathies, Coronary Heart Disease, Heart Failure, Myocardial infarction and Peripheral Vascular Disease.
