# Supplementary material for: Survival improvement for patients with metastatic colorectal cancer over twenty years
Source: NPJ Precis Oncol. 2023 Feb 13;7:16. doi: 10.1038/s41698-023-00353-4 (PMC9925745; doi:10.1038/s41698-023-00353-4)
Supplement: Supplementary file 2 — REPORTING SUMMARY [file 41698_2023_353_MOESM2_ESM.pdf]

nature portfolio

Corresponding author(s): John Paul Shen

Last updated by author(s): Nov 17, 2022

Reporting Summary

Nature Portfolio wishes to improve the reproducibility of the work that we publish. This form provides structure for consistency and transparency in reporting. For further information on Nature Portfolio policies, see our Editorial Policies and the Editorial Policy Checklist.

Statistics

For all statistical analyses, confirm that the following items are present in the figure legend, table legend, main text, or Methods section.

n/a	Confirmed	
☐	☒	The exact sample size (*n*) for each experimental group/condition, given as a discrete number and unit of measurement
☐	☒	A statement on whether measurements were taken from distinct samples or whether the same sample was measured repeatedly
☐	☒	The statistical test(s) used AND whether they are one- or two-sided<br>*Only common tests should be described solely by name; describe more complex techniques in the Methods section.*
☐	☒	A description of all covariates tested
☐	☒	A description of any assumptions or corrections, such as tests of normality and adjustment for multiple comparisons
☐	☒	A full description of the statistical parameters including central tendency (e.g. means) or other basic estimates (e.g. regression coefficient) AND variation (e.g. standard deviation) or associated estimates of uncertainty (e.g. confidence intervals)
☐	☒	For null hypothesis testing, the test statistic (e.g. *F*, *t*, *r*) with confidence intervals, effect sizes, degrees of freedom and *P* value noted<br>*Give P values as exact values whenever suitable.*
☒	☐	For Bayesian analysis, information on the choice of priors and Markov chain Monte Carlo settings
☒	☐	For hierarchical and complex designs, identification of the appropriate level for tests and full reporting of outcomes
☐	☒	Estimates of effect sizes (e.g. Cohen's *d*, Pearson's *r*), indicating how they were calculated

Our web collection on statistics for biologists contains articles on many of the points above.

Software and code

Policy information about availability of computer code

Data collection	Patients were identified using the Foundry software system (Palantir Technologies, Denver, CO).
Data analysis	All analyses were performed using SPSS version 26.0 (SPSS, Chicago, IL), and GraphPad Prism version 8.0 (GraphPad; La Jolla, CA), and R version 4.0.1

For manuscripts utilizing custom algorithms or software that are central to the research but not yet described in published literature, software must be made available to editors and reviewers. We strongly encourage code deposition in a community repository (e.g. GitHub). See the Nature Portfolio guidelines for submitting code & software for further information.

Data

Policy information about availability of data

All manuscripts must include a data availability statement. This statement should provide the following information, where applicable:

- Accession codes, unique identifiers, or web links for publicly available datasets
- A description of any restrictions on data availability
- For clinical datasets or third party data, please ensure that the statement adheres to our policy

The datasets generated during and/or analyzed during the current study are not publicly available to maintain compliance with IRB protocol. Anonymized data are available from corresponding author upon request.

Human research participants

Policy information about studies involving human research participants and Sex and Gender in Research.

Reporting on sex and gender	Gender was determined based on self-reporting by patient available in EHR. We did not determine or include the sex of the patient in our manuscript.
Population characteristics	Age at diagnosis, Anti-BRAF, Anti-EGFR, Cardiovascular, Chronic Kidney Disease, Depression, Diabetes, Gender, Hyperlipidemia, Hypertension, Immunotherapy, Liver Resection, Third Line Treatment, Race (Asian/ Black or African American/ Hispanic or Latino), Primary Tumor Sidedness, Thyroid, Year of Diagnosis.
Recruitment	Adult patients diagnosed with metastatic CRC were identified from the electronic health record (EHR) at the University of Texas M.D. Anderson Cancer Center (UTMDACC) using the Foundry software system (Palantir Technologies, Denver, CO). Patients diagnosed between January 1, 2004, and December 31, 2019, were selected for study, with follow-up until April 15, 2021. Only patients with confirmed adenocarcinoma of colon and/or rectum and de novo metastatic disease were included in the study.
Ethics oversight	IRB of The University of Texas M.D. Anderson Cancer Center

Note that full information on the approval of the study protocol must also be provided in the manuscript.

Field-specific reporting

Please select the one below that is the best fit for your research. If you are not sure, read the appropriate sections before making your selection.

☒ Life sciences ☐ Behavioural & social sciences ☐ Ecological, evolutionary & environmental sciences

For a reference copy of the document with all sections, see nature.com/documents/nr-reporting-summary-flat.pdf

Life sciences study design

All studies must disclose on these points even when the disclosure is negative.

Sample size	No sample-size calculation was done. Adult patients diagnosed with metastatic CRC were identified from the electronic health record (EHR) at the University of Texas M.D. Anderson Cancer Center (UTMDACC) using the Foundry software system (Palantir Technologies, Denver, CO). Patients diagnosed between January 1, 2004, and December 31, 2019, were selected for study, with follow-up until April 15, 2021. Only patients with confirmed adenocarcinoma of colon and/or rectum and de novo metastatic disease were included in the study. Patients who visited just for consultation and did not receive their treatment at UTMDACC were excluded
Data exclusions	Patients who visited just for consultation and did not receive their treatment at UTMDACC were excluded. Non-metastatic CRC patients were also excluded.
Replication	This is a retrospective analysis of patients datasets available in our institute's repository. We did not perform any prospective analysis to reproduce our results or replicate the same analysis in another dataset. Due to nonavailability of a similar cohort with the same information of patients, this study could not be replicated.
Randomization	No randomization was done for this study as this is a retrospective study.
Blinding	No blinding was done for this study as this is a retrospective study.

Reporting for specific materials, systems and methods

We require information from authors about some types of materials, experimental systems and methods used in many studies. Here, indicate whether each material, system or method listed is relevant to your study. If you are not sure if a list item applies to your research, read the appropriate section before selecting a response.

Materials & experimental systems

n/a	Involved in the study
☒	☐ Antibodies
☒	☐ Eukaryotic cell lines
☒	☐ Palaeontology and archaeology
☒	☐ Animals and other organisms
☐	☒ Clinical data
☒	☐ Dual use research of concern

Methods

n/a	Involved in the study
☒	☐ ChIP-seq
☒	☐ Flow cytometry
☒	☐ MRI-based neuroimaging

Clinical data

Policy information about clinical studies

All manuscripts should comply with the ICMJE guidelines for publication of clinical research and a completed CONSORT checklist must be included with all submissions.

Clinical trial registration	This is a retrospective analysis and not a clinical trial.
Study protocol	Lab 09-0373
Data collection	Adult patients diagnosed with metastatic CRC were identified from the electronic health record (EHR) at the University of Texas M.D. Anderson Cancer Center (UTMDACC) using the Foundry software system (Palantir Technologies, Denver, CO). Patients diagnosed between January 1, 2004, and December 31, 2019, were selected for study, with follow-up until April 15, 2021.
Outcomes	N/A
